# Why NHS hospital co-morbidity research may be wrong: how clinical coding fails to identify the impact of diabetes mellitus on cancer survival

**DOI:** 10.1038/s41416-025-03136-9

**Published:** 2025-08-09

**Authors:** K. Zucker, C. McInerney, A, Glaser, P. Baxter, G. Hall

**Affiliations:** 1 Leeds Institute of Data Analytics, University of Leeds, Leeds, UK; 2 School of Medicine, University of Leeds, Leeds, UK; 3 Leeds Teaching Hospitals NHS Trust, Leeds, UK; 4 Sheffield School for Health and Related Research (SCHARR), University of Sheffield, Sheffield, UK

**Keywords:** Cancer, Biostatistics, Oncology, Diabetes, Cancer epidemiology

## Abstract

**Background:**

Significant volumes of research rely on secondary care diagnostic coding to identify comorbidities however little is known about its accuracy at a population level or if this influences subsequent analysis.

**Methods:**

Retrospective observational study utilising real world data for all cancers, prostate cancer and breast cancer patients diagnosed at Leeds Cancer Centre from 2005 and 2018. Three different data definitions were used to identify patients with diabetes in each cohort: (1) clinical coding alone, (2) HbA1c blood test alone (3) either clinical coding or abnormal HbA1c. Cohort characteristics, diagnosis dates and Cox derived survival was compared across diabetes definitions.

**Results:**

123,841 cancer patients were identified including 13,964 with diabetes. Clinical coding failed to identify 14.6% of diabetic cancer patients with a temporal misclassification rate of 17.5%. Sole reliance on clinical coding overestimated the negative effect of DM on median survival across all cancers and 3.17 years in breast cancer.

**Discussion:**

Clinical coding provides inaccurate diabetes diagnosis date and detection resulting in meaningful differences in analytic outcomes. This supports the use of more detailed comorbidity data definitions. Results casts doubt over research reliant on hospital clinical coding alone and the generalisability of some comorbidity and frailty scoring systems.

## Introduction

With an aging population globally, particularly in more economically developed countries, a greater percentage of the population are living with significant health problems [1, 2]. As a result, the issues of frailty, comorbidity and multi-morbidity are areas of not only growing clinical importance, but the focus of intensive research and scientific interest [3, 4]. Much work has focussed on how specific comorbidities or constellations of comorbidities impact health outcomes, with previous literature focussing on disease specific outcomes or survival [5].

Research in this area is not possible via a randomised controlled trial, as patients cannot be randomised to the pre-existing health condition of interest. Hence, research frequently involves retrospective analyses of routinely collected data or prospective cohort and case control studies. A challenge when conducting retrospective analyses is the identification of the conditions of interest from a patients’ electronic health record without the need for manual review. Within the United Kingdom (UK), many researchers make use of clinical coding for this task. Clinical coding is administrative data that is generated every time a patient is admitted to hospital [6]. Each admission is assigned an ICD-10 code [7] for the main cause of admission and further secondary ICD-10 codes for each other condition relevant to the admission or that the patient is known to have. This coding is performed by specially-trained clinical coders, who review the clinical documentation and input the information. English hospitals submit these data to NHS England [8], a national organisation aiming as part of their remit to use digital technology to improve delivery of health and social care in England, who combine all the results from all of the English hospitals to form the Hospital Episode Statistics Dataset (HES) [6]. Outpatient events are found in this dataset, however these episodes do not have accompanying diagnostic clinical coding data. HES coding data is therefore perhaps better described by the phrase ‘post-discharge administrative coding’.

Hospital clinical coding and the HES datasets [6] are widely used for research and the data is commonly included in or used alongside other nationally collated datasets, such as the UK national cancer registry [9]. As a country wide, centrally curated dataset it potentially offers a simple approach to identify patients with comorbidities of interest. Consequently significant volumes of research have been published based on HES data: in many cases attempts have been made to evaluate the impact of comorbidity on outcomes in a number of settings including renal medicine [10], general surgery [11], urology [12] and oncology [13, 14]. Despite its widespread use, including within the NHS England Secure Development Environemt [15], the accuracy of clinical coding has been called into question [16–21] with one study highlighting diabetes mellitus as a particular problem area [22].

Previous research assessing the accuracy of clinical coding has mainly been conducted in the area of surgery [16, 18, 20, 21] and focussed on coding after a specific admission event. These studies have universally identified issues with the accuracy of clinical coding in particular issues around poor negative predictive value [21]. A large scale audit of over 30,000 surgical patient records found coding errors in 51% of patient admissions [18]. Given the significant body of evidence questioning the accuracy of clinical coding after a hospital admission we set out to assess how well clinical coding affected the identification of diabetes mellitus in the overall oncology population which includes both patients with and without hospital admissions. Little is known about how well clinical coding captures disease in the wider patient population that includes patients who are managed and diagnosed wholly or in part on an outpatient basis, and whether there is a greater issue of missed coding in this group of patients. This is of particular relevance to specialties that manage patients mainly on an outpatient basis, where comorbidities may never be coded. Patients with missing clinical coding might systematically differ from both their coded counterparts and their truly-code free counterparts. The results from comparative analyses of these patient groups could differ depending on how the non-coded patients are assigned. We aim to quantify the fidelity of clinical coding for the identification of cancer patients with diabetes mellitus by comparing these to different data definitions of diabetes. We also quantify how this influences cohort size, estimated date of diagnosis and survival estimates for cancer patients with diabetes. This study is not intended or designed to yield results to describe the true relationship between diabetes and cancer outcomes, it instead uses this analysis as a means of assessing and informing the validity and clinical utility of diabetes defined by hospital administrative data alone for comorbidity and risk score based research in the UK.

## Methods

### Dataset

We studied the routinely-collected healthcare records of patients in the Patient Pathway Manager (PPM) system used by the Leeds Teaching Hospitals NHS Trust (LTHT). Only patients having a legitimate care relationship with Leeds Cancer Centre who were diagnosed with cancer between 2005-2018 were included. All data was analysed within secure NHS infrastructure complying with ISO 27100 and NHS Data Security and Protection Toolkit. The dataset was anonymised and underwent data obfuscation, e.g. age presented in 5-year age bands prior to release for analysis.

### Identifying cancer diagnoses

We identified patient records with a definitive primary diagnosis of malignancy by searching for ICD-10 ‘C’ codes. Where records showed more than one primary cancer diagnosis, the earliest was selected and later diagnosis excluded. Information relating to patients’ cancer diagnosis, demographics, clinical coding and HbA1c blood results were extracted. Separate populations for breast cancer and prostate cancer were extracted based on cancer specific ICD-10 codes from the overall PPM population. Where patients had multiple diagnoses with the same cancer the earliest was selected and later diagnosis excluded. Breast and Prostate cancer were chosen as exemplars due to their high incidence and relatively long median survival as compared to other malignancies.

### Identifying diabetes mellitus

Diabetes mellitus (hereon referred to as diabetes) can be indicated by clinical coding or abnormal HbA1c results. All clinical coding events were analysed to identify any instance of a diabetic ICD-10 code within their coded events (see Supplementary File 1 for ICD-10 code diabetes data definitions). The earliest date of coding was taken as the diagnosis date for diabetes. Patients with HbA1c results of 48 mmol/mol or above were also identified as diabetic, with the earliest abnormal HbA1c results taken as the date of diagnosis. Older results recorded using percentage values had previously been converted to mmol/mol prior to analysis. This threshold was chosen to be in line with international diagnostic guidelines [23]. We define three identification methods of diabetes: abnormal HbA1c, clinical coding and a hybrid of either abnormal HbA1c or clinical coding. In the case of the hybrid approach, if a patient had both abnormal bloods and clinical coding, then the earlier of these two events was treated as the diabetes diagnosis event.

### Analysis of patient characteristics

**Fig. 1:**
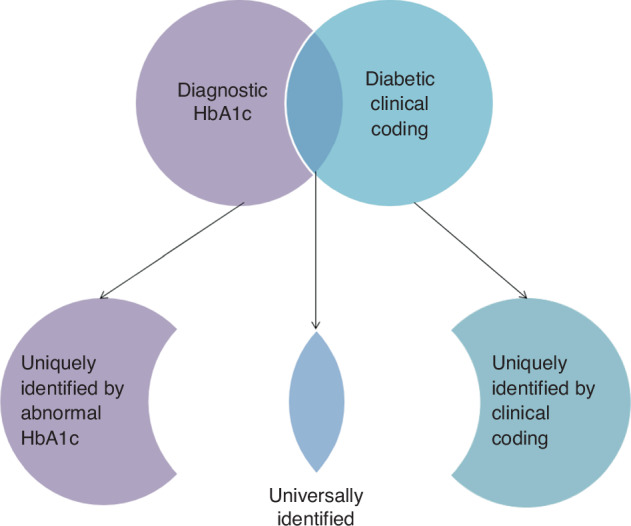
Graphical representation of the diabetic subgroups. Note that the shape area of each subgroup is not scaled to the true numbers in the dataset.

Comparison of baseline characteristics was conducted to identify any systematic differences between patients identified HbA1c but not clinical coding (uniquely identified by HbA1c), patients identified by clinical coding but not HbA1c (uniquely identified by clinical coding) and those universally identified i.e. identified by clinical coding and HbA1c (Fig. 1). Given strong violation of normality assumptions, Mann-Whitney U tests were conducted between pairs of sub-groups to test for non-difference of the distributions for age, sex (excluding prostate cancer group) and deprivation levels. A 5% level of significance was applied for these comparisons.

### Temporal analysis

To assess whether clinical coding defined diabetes provides an accurate surrogate marker for diabetes diagnosis date, we calculated the time lag between the estimated timing of diagnosis generated from clinical coding and the estimate generated from HbA1c blood results in patients that had been diagnoses by both clinical coding and HbA1c. Temporal misclassification error was calculated for patients identified as having post-cancer diabetes by clinical coding. This was achieved by identifying the percentage of patients that were identified as having post-cancer diabetes by clinical coding that were identified as having pre-cancer diabetes by the hybrid definition across the three cohorts.

### Survival analysis

Survival analysis was conducted using Cox proportional hazard adjusting for age, sex (excluding prostate cancer group) and Index of Multiple Deprivation (IMD) quintile. Four Cox models were built per cancer cohort one for the full population of patients including those with and without diabetes, and one for each of the populations of patients identified as diabetic by the three data definitions. The resultant survival trajectories were compared across the dataset visually with survival curves, and comparisons of median survival for clinical significance. Differences in estimated median survival between the 3 diabetes definition models of over 6 months was deemed clinically meaningful, which was based on local expert opinion. We did not test whether the survival estimates for each group showed a statistically significant difference between one another as the populations have incomplete pairing. This is due to the partial overlap of cohorts identified by each diabetic cohort with some patients appearing in multiple diabetic cohorts and some in only one. All analyses were undertaken using R (version 4.2.1) and open-source packages available on CRAN.

Results were represented by hazard ratios attributable to diabetes derived from the model coefficients, and the percentage change in median overall survival between each of the diabetic subgroups and non-diabetic groups. Those where the estimated hazard ratio and its associated 95% confidence intervals did not include 1 were deemed to be meaningful results because they have a high confidence that the hazard is unidirectional.

### Sensitivity analysis

To mitigate for a potential boundary effect introduced by blood lab geographical boundaries, a sub-population of patients was created to include only those patients living within the area for which LTHT’s blood lab analyses primary care blood samples, which we have termed the ‘LTHT blood catchment area’. This area was defined by patients being registered to a general practice that had provided over 10,000 previous blood samples to LTHT. This cut off was chosen based on local expert opinion. The date of diabetes diagnosis as per the three definitions was compared to the date of cancer diagnosis. Those patients with a diabetic diagnosis date on or before the date of cancer diagnosis were treated as patients with pre-existing diabetes.

## Results

A total of 123,841 unique patients were identified, including 17,920 breast cancer patients and 15,856 prostate cancer patients. A total of 20,589 patients had more than one malignancy diagnosis representing 16.6% of the total patient population. Between 14.6 and 21.3% of total diabetic patients identified by the hybrid definition were not identified when only clinical coding was used across the cohorts. A greater proportion were missed when HbA1c was used alone with between 15.7 and 27.0% failing to be identified.

### Baseline characteristics

Table 1 presents the comparison of baseline characteristics between the three sub-groups that constitute the hybrid definition of diabetes. Pairwise statistical significance shown in Table 2 identifies that diabetic patients identified by abnormal HbA1c and not clinical coding were younger in all cohorts. Statistically significant differences seen in other parameters were not consistent across cohorts.

**Table 1:** Count of patients by each diabetes identification method.

Cohort	Diabetes Definition	Pre-Cancer	Post-Cancer	Total
All	Coding	5849 (72.7%)	6080 (102.8%)	11,929 (85.4%)
	HbA1c	6149 (76.4%)	4042 (68.4%)	10,191 (73.0%)
	Hybrid	8051	5913	13,964
Breast	Coding	423 (61.4%)	600 (98.2%)	1023 (78.7%)
	HbA1c	577 (83.7%)	519 (84.9%)	1096 (84.3%)
	Hybrid	689	611	1300
Prostate	Coding	546 (62.6%)	824 (98.7%)	1370 (80.3%)
	HbA1c	715 (82.0%)	627 (75.1%)	1342 (78.6%)
	Hybrid	872	835	1707

Percentages are expressed relative to the total number of patients identified by the hybrid definition of diabetes.

**Table 2:** Baseline characteristics of diabetic patients.

Cohort	Variable	Uniquely Identified by HbA1c	Uniquely Identified by Coding	Universally Identified
All	Median Age (Years)	70–74	70–74	70–74
	Median IMD	3	2	2
	Percentage Female	41.45%	37.79%	41.51%
Breast	Median Age (Years)	65-69	75–79	70–74
	Median IMD	2	2	2
	Percentage Female	99.24%	98.21%	99.35%
Prostate	Median Age (Years)	70–74	75–79	70–74
	Median IMD	3	3	3

Comparison is made between the different manner in which they could be included in the hybrid diabetic defined cohort. Patients might be identified by coding but not HbA1c, HbA1c but not coding or identified universally (by both). Note that average age is represented as a range as per the source data.

### Sensitivity analysis

**Fig. 2:**
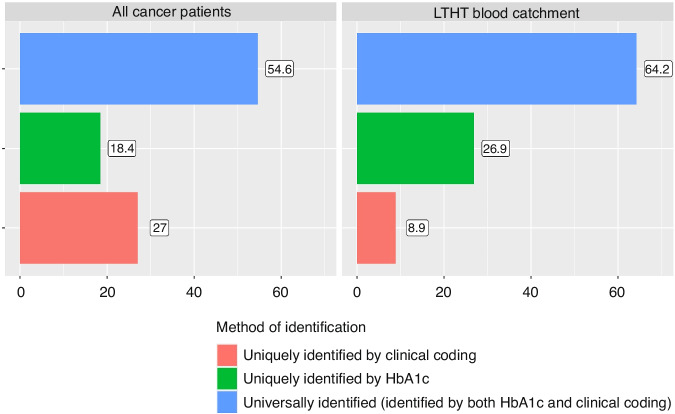
Overlap of identification by HbA1c and clinical coding. Proportion of the diabetic cancer population that are identified by Clinical coding but not HbA1c (Uniquely Identified by Clinical Coding), HbA1c and not clinical coding (Uniquely Identified by HbA1c), or identified by both HbA1c and Clinical Coding (Universally Identified). The results are shown for all cancer patients and the population of cancer patients in the LTHT blood catchment area.

Figure 2 shows the diabetic cohort as identified by the hybrid definition, broken down by the data indications for diabetes contained in patient’s records: coding only, abnormal HbA1c only, both coding and abnormal HbA1c. Records for patients in both the full and the LTHT-blood-catchment-area cohort predominantly contained both indicators for a diabetic diagnosis. Of the remaining patients with records of either coding or abnormal HbA1c, the LTHT-blood-catchment-area cohort were indicated by abnormal HbA1c markedly more often than in the full cohort. This pattern was consistent across cancer sites.

### Temporal analysis

**Fig. 3:**
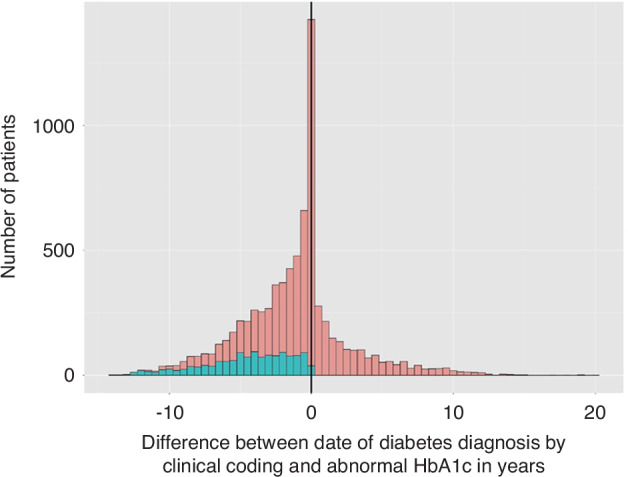
Difference in first diabetic diagnosis flag from clinical records comparing clinical coding and abnormal HbA1c. = patients defined by both an abnormal HbA1c and diabetic clinical coding.  = patients defined by clinical coding as post-cancer diabetics but with abnormal HbA1c pre-cancer. Left of  = abnormal HbA1c earlier than clinical coding. Right of  = clinical coding earlier than abnormal HbA1c.

Table 3 shows a breakdown of the number of diabetic patients identified before and after cancer diagnosis in each cancer cohort. Clinical coding demonstrated a temporal misclassification rate of 17.5% in the all cancer cohort 25.2% of the breast cancer cohort and 22.3% of prostate cancer cohort. The high rates of post-cancer diabetic patients identified by clinical coding only is partly due to differences in timing of first evidence of a diabetic diagnosis when comparing abnormal HbA1c results to clinical coding. Figure 3 highlights differences between the clinical-coding and HbA1c-results approaches to diabetes identification, some of which are greater than ±15 years.

**Table 3:** Results from Pairwise Comparison of Baseline Characteristics using the Mann-Whitney *U* test—*p* values generated by comparing the patients uniquely identified by HbA1c testing to those uniquely identified by clinical coding and those universally identified.

Cohort			Coding	Universally Identified
All	Age	Coding		*p* < 0.01
		HbA1c	0.01	*p* < 0.01
	Sex	Coding		0.01
		HbA1c	0.02	0.97
	IMD	Coding		0.2
		HbA1c	*p* < 0.01	*p* < 0.01
Breast	Age	Coding		0.57
		HbA1c	*p* < 0.01	*p* < 0.01
	Sex	Coding		0.93
		HbA1c	0.95	0.97
	IMD	Coding		0.59
		HbA1c	0.12	0.19
Prostate	Age	Coding		0.67
		HbA1c	*p* < 0.01	*p* < 0.01
	IMD	Coding		0.6
		HbA1c	0.39	0.65

The uniquely identified by clinical coding group was also compared to the universally identified group. The table presents the variable of interest in the first column the first group in the pairwise comparison in the second column, the results are presented in the third and fourth column and the last column represents the cohort for analysis. The two labels above the third and fourth column represent the second comparator group used in the pairwise test.

### Survival analysis

**Fig. 4:**
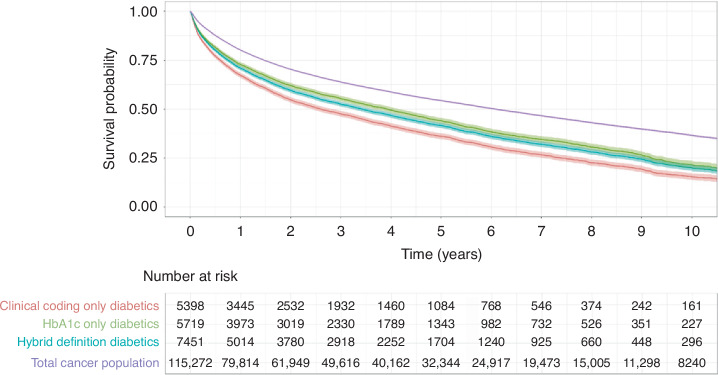
Survival trajectories for each diabetic data definition and the overall survival for all patients in the all cancer cohort. Survival curves have been plotted for cox models generated using only all patients, coded diabetic patients, abnormal HbA1c patients and diabetic patients identified by the hybrid method. In all cases estimates were adjusted for potential age, sex and deprivation confounding.

**Fig. 5:**
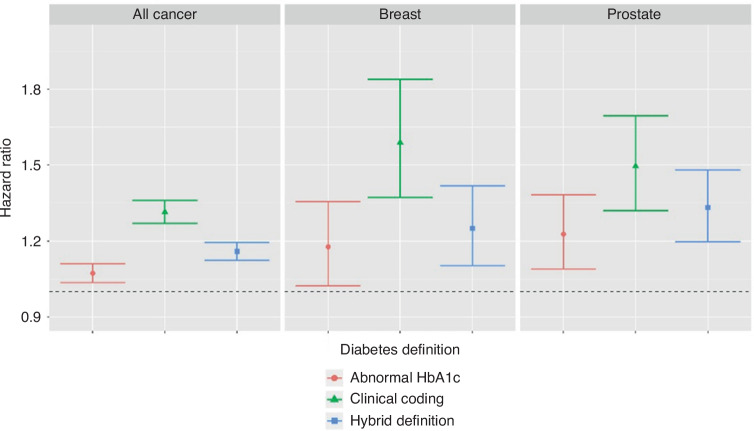
Cox derived hazard ratios for the impact of diabetes. Comparison between the estimated hazard across all three cohorts using each of the diabetes definitions. Horizontal dashed line indicates a hazard ratio of 1.0, which is the threshold at which the estimated hazard is equal with and without diabetes

Assessment of the survival difference for all cancer patients demonstrates clinically meaningful differences in the survival estimates obtained via each of the methods for identifying diabetes (Fig. 4, Table 4). In all three diabetic data definitions, the overall survival outcome for diabetic patients is worse than that of the overall survival trajectory for all patients. Patients identified by abnormal HbA1c as the sole definition demonstrate the most optimistic diabetic survival trajectory, while patients identified by clinical coding as the sole definition demonstrated the most pessimistic. The survival curve for patients identified by the hybrid definition lies in between the others. This pattern of difference was consistent across all three cancer cohorts analysed (Fig. 5).

**Table 4:** Comparison of Median Survival Estimates for Each Diabetes.

Cohort	Diabetic Data Definition	Median Survival (Years)	Survival Difference from baseline (Years)
All	Clinical Coding	2.61	−3.48
	Abnormal HbA1c	3.92	−2.17
	Hybrid	3.42	−2.67
Breast	Clinical Coding	5.51	−7.06
	Abnormal HbA1c	9.03	−3.54
	Hybrid	8.14	−4.43
Prostate	Clinical Coding	5.49	−4.80
	Abnormal HbA1c	7.57	−2.73
	Hybrid	6.89	−3.40

Definition—Median survival estimates were extracted from each of the Cox models. The difference from baseline overall median survival for both diabetic and non-diabetic patients in each cancer cohort is also calculated and presented.

## Discussion

In this study, we assessed the fidelity of clinical coding at a population level and how clinical coding omissions affect the results of analysis. We found that survival estimates for cancer patients with diabetes mellitus are more pessimistic when diabetes mellitus is informed by hospital clinical coding alone compared to HbA1c levels and a hybrid approach. This finding is of particular relevance to clinical decision making based on automated risk-scoring algorithms and to specialties that manage patients mainly on an outpatient basis.

Our data identifies a meaningful proportion of diabetic patients in the all-cancer cohort whose date of diabetes diagnosis is incorrectly indicated temporally by clinical coding or completely absent. Clinical Coding failed to identify 18.4% of all diabetic cancer patients, which is similar to the 14% error rate for diabetic clinical coding identified in the literature [20]. The LTHT blood catchment area cohort identifies a larger 26.9% with missing diabetic coding. This may suggest that in the full LTHT dataset we are still failing to identify a cohort of diabetic patients who may have been identified on blood testing where the results are held by other hospitals across the region and thus are not included within the hospitals blood dataset. If these patients could be identified, then the results of subsequent analyses may show even greater levels of outcome discrepancy than seen in our results.

Through the use of HbA1c blood tests, we were able to create a second check for a diabetes diagnosis in a patient’s clinical record. This enabled us to assess the fidelity of clinical coding and identify whether the wider data definition and resulting populations impacted upon survival analysis outcomes. The missed diabetic patients that are identified by abnormal HbA1c values differ significantly in baseline characteristics from the identified cohort.

A large minority of those patients identified by both blood test results and clinical coding are identified at a much later date than their first diagnostic blood test result. Whilst the raw numbers identified by clinical coding as having diabetes which developed after their cancer diagnosis look largely comparable to the hybrid definition (Table 2), this is a function of the high rates of temporal misclassification error. Of the 6080 patients identified in the all-cancer cohort, 1138 in fact had evidence of diabetes on blood results which predated their cancer diagnosis. This accounts for why the number of patients in the post-cancer period identified by clinical coding exceed the number identified by the hybrid definition.

For an analysis based on the presence or absence of diabetes at an index date – in our case cancer diagnosis – the discrepancy due to the choice of diabetic data definition could have a profound impact on the correct identification of the comorbid cohort. Identifying cohorts of patients with diabetes using hospital clinical coding as the sole method of identification has a high risk of misclassifying patients as non-diabetic at the index date.

Our results show that the differences in the cohort identification does not merely alter the comorbid cohort size and precision, but additionally affects the analysis results. The difference seen in projected hazard and median survival are substantial and clinically meaningful. The results show that the incorporation of blood results into defining a diabetic population increases the number of patients identified, improves temporal accuracy and alters the analysis output. Further survival analyses and commentary on this can be found within Supplementary File 2. It additionally highlights that the clinical coding-only diabetic cohort differs from the wider diabetic cohort as defined by the hybrid data definition. This suggests that the assumption that comorbidity scores, developed and validated on administrative data, will be equally valid when applied to the general population may not hold. Although large meaningful error rates have been identified in previous research, the impact on analysis outcome has never previously been assessed.

Research attempting to assess how comorbidity impacts on outcomes and risks have commonly relied on hospital clinical coding and similar administrative datasets. Several ubiquitous comorbidity scores such as Charlson Score [24] and the Elixhauser [25] have been developed on administrative data and are subsequently being used in clinical practice based on comorbidity data obtained from referral letters, clinical records and the patient directly. In such cases, clinicians are working on the assumption that administrative comorbidity data is representative of the true comorbid population. Our results suggest that this assumption in the case of diabetes is incorrect and using risk scores in this manner may result in incorrect risk being ascribed to patients. This may explain the variability in the estimated utility of these tools across the literature particularly in the cancer population, where identifying superiority of one scoring system over others has remained elusive [26–28].

Within our analysis the hybrid definition is used as the reference standard against which fidelity was assessed. This however is not the true gold standard which would require manual curation and review of patient records from all healthcare settings. In most instances however doing this at scale is not practicable due to cost, time and data privacy constraints. As such alternatives making best use of available data will continue to be used in real world evidence studies.

Previous literature has identified a large number of conditions beyond diabetes that demonstrate coding inaccuracy [21] and thus diabetes might not be the only condition to demonstrate differences between hospital administrative data and the true generally comorbid population. Our results demonstrate the benefit of defining comorbidity in a more comprehensive manner than relying purely on hospital clinical coding. The results comparing the fidelity of coding for patients inside and outside the ‘LTHT blood catchment area’ highlight the issue that data fragmentation and siloing causes. Even where data definitions may be enhanced through more diverse data items, ensuring robust and consistent capture and coverage of this data including across geographies is essential to avoid introducing other sources of bias which may also limit the generalisability of the resulting analytical outputs.

In this study, we have focussed on blood results because there is a clear and reliable diagnostic test-based definition for diabetes; but similar diagnostic tests are not available for many other conditions. In such cases, hospital electronic health records could be enhanced with other indicative data such as primary-care coding [29], prescribing data and natural language processing of free text clinical narrative. As demonstrated with the addition of the HBA1C data which highlighted significant geographical variation in its utility, these further data sources cannot be added blindly and requires significant thought and investigation so as to consider, and where possible, take into account the additional biases they may introduce.

Our study data is derived from a single NHS trust and the quality and accuracy of clinical coding might differ between hospitals around the country. This data was derived from diagnostic codes entered by clinical coders which occurs after an admission event. Some centres may have more advanced record keeping systems which allow for the clinical professionals to directly enter diagnostic codes. These datasets may therefore be more accurate in both the number of patients and the timing of diagnosis. Clinical coder derived data however forms the basis of HES which remains a significant and often solitary data source in the literature for the identification comorbidity on English national data. Many of the risk scores highlighted above have been developed on US data, which might differ in other ways, too. Further research is needed to identify whether the same cohort identification and outcome differences are seen elsewhere in the UK and abroad. Incorporating data from primary care may alter the data accuracy and further study using linked data is therefore required to assess this. The difficulty with this is that blood test results are not always available alongside the administrative data, particularly in registry or claims-based datasets. We hypothesise that records of diabetic patients without clinical coding occurs due to patients who do not have hospital admissions since their diabetic diagnosis. If this is the case, then the results we have identified are likely to be widespread rather than local.

Whilst the results presented focus on diabetes in a cancer population as an exemplar, this was not the focus of our analysis, which has been designed to assess the fidelity hospital derived clinical coding as a source for comorbidity data. It does not therefore represent a comprehensive assessment of the impact of diabetes on cancer outcomes. Further research focussing on this question using net survival, cause specific hazards and other analytics techniques would be required to robustly estimate this complex outcome question.

Our analysis makes adjustment for confounding based on age, sex (where relevant) and deprivation; however further sources of confounding may be present. It might therefore be possible that further appropriate controlling of confounders could account for divergence of projected hazards among the different diabetic cohorts. Within our dataset, additional adjustment for grade, stage and histology resulted in the same pattern of outcomes being identified. These were not included in the main results as both grade and stage definitions differ both over time and between cancer sites and data completeness was inconsistent. Our study only includes patients diagnosed up until the end of 2018. This was designed to ensure that patients would have had a sustained period of follow up prior to the COVID-19 pandemic. This does raise the possibility of changes in practice and clinical coding since then which will not be captured by our analysis.

Overall, our results add to the body of literature highlighting significant omissions in clinical coding data. To the best of our knowledge, we demonstrate for the first time the scale of limitations of clinical coding at a population level where accuracy is not only assessed on patients with an admission, but also patients without a recent admission or no admission at all. Furthermore, our work highlights that the coding inaccuracy leads to meaningful differences in analysis results.

## Conclusion

Serious questions are raised about hospital clinical coding and its utility in diabetic comorbidity research given the substantial differences demonstrated in projected hazard from the inclusion of a more comprehensive data definition. If the pattern of identification of other comorbidities via hospital clinical coding is similar to that of diabetes, then it is possible that clinical coding and derived datasets significantly underrepresents health problems. Furthermore, clinical coding might include patients with worse outcomes when compared to the non-coded comorbid population, as is the case in patients with diabetes. Consequently, it is likely that results generated utilising hospital clinical coding will be different from those found in the wider general population and should not be used in routine clinical decision making unless specifically validated for this use case. More research is required to assess the reproducibility of HES and clinical coding derived results in the general population and to identify whether current and future tools based upon these are being applied safely and appropriately.

This research lends weight to argument for a move from a reliance solely on hospital clinical coding for defining comorbidity and the use of more detailed datasets that include a greater ability to identify diagnoses of interest via multiple routes such as from primary care coding, investigation results and analysis of free text.

## Supplementary information

Table of Diabetes Code Definitions

Further Survival Analysis

## Data Availability

This analysis was undertaken within LTHT systems. In view of the patient level data used it will not be released for general use. LTHT does have a formal mechanism for applying for data access and release, the details of which can be found at: www.leedsth.nhs.uk/research/our-research/#:~:text=Prior%20to%20any%20release%20of,for%20details%20of%20these%20courses.

## Code availability

R code for the analysis can be found online at: https://github.com/kieranzu/HbA1c.
