# Supplementary material for: Why NHS hospital co-morbidity research may be wrong: how clinical coding fails to identify the impact of diabetes mellitus on cancer survival
Source: Br J Cancer. 2025 Aug 9;133(8):1137–44. doi: 10.1038/s41416-025-03136-9 (PMC12532788; doi:10.1038/s41416-025-03136-9)
Supplement: Supplementary file 1 — Table of Diabetes Code Definitions [file 41416_2025_3136_MOESM1_ESM.docx]

**Supplementary File 1**

| **ICD** | **Description** | **Comments** |
| --- | --- | --- |
| E10 | Type 1 Diabetes Mellitus | Includes subsets 0-9 |
| E11 | Type 2 Diabetes | Includes subsets 0-9 |
| E12 | Malnutrition-related diabetes mellitus | Includes subsets 0-9 |
| E13 | Other Specified diabetes mellitus | Includes subsets 0-9 |
| E14 | Unspecified Diabetes Mellitus | Includes subsets 0-9 |
| O24.0 | Diabetes Mellitus in Pregnancy: Pre-existing type 1 diabetes Mellitus |  |
| O24.1 | Diabetes Mellitus in Pregnancy: Pre-existing type 2 diabetes mellitus |  |
| O24.2 | Diabetes Mellitus in Pregnancy: Pre-existing malnutrition related diabetes mellitus |  |
| O24.3 | Diabetes Mellitus in Pregnancy: Pre-existing diabetes mellitus unspecified |  |
| G59.0 | Diabetic mononeuropathy |  |
| G63.2 | Diabetic polyneuropathy |  |
| H28.0 | Diabetic cataract |  |
| H36.8 | Diabetic retinopathy |  |
| I79.2 | Peripheral angiopathy in diseases classified elsewhere |  |
| M14.2 | Diabetic arthropathy |  |
| N08.3 | Glomerular disorders in diabetes mellitus |  |

Table 1: ICD-10 code definitions used to identify diabetic patients from clinical coding
