# Supplementary material for: Why NHS hospital co-morbidity research may be wrong: how clinical coding fails to identify the impact of diabetes mellitus on cancer survival
Source: Br J Cancer. 2025 Aug 9;133(8):1137–44. doi: 10.1038/s41416-025-03136-9 (PMC12532788; doi:10.1038/s41416-025-03136-9)
Supplement: Supplementary file 2 — Further Survival Analysis [file 41416_2025_3136_MOESM2_ESM.docx]

**Supplementary File 2**

# **Introduction:**

Although providing a definitive and complete analysis of the survival outcomes for patients with both diabetes mellitus and a cancer diagnosis was not the aim of this manuscript, reviewers suggested that adding in further analysis would be beneficial to the work presented within the main body of the manuscript. We have therefore undertaken the additional recommended analysis and present this with stand-alone sections detailing the methods, results and discussions for these additional experiments.

# **Methods:**

## **Survival Analysis:**

As within the main body of the paper survival analysis was undertaken using Cox proportional hazards.^1^ Models were built for each of the definitions of diabetes mellitus with adjustments made for age, sex (where relevant) and Index of Multiple Deprivation.^2^ For each model the effect of diabetes was extracted from the model with results compared across the three diabetes data definitions. Analyses were run for each of the cohorts described below to include all cancer patients, prostate cancer and breast cancer.

## **Additional Cohorts:**

### Leeds Teaching Hospitals NHS Trust (LTHT) Blood Catchment Area Cohort:

This cohort was the same as was defined within the main body of the paper. It includes all cancer patients who were known to be registered to a GP that was within the Leeds Teaching hospital blood lab catchment areas which was defined as GP practices that had 10,000 or more sample received by the LTHT laboratory

### Admitted Cohort:

A further cohort was created which included only cancer patients with an admission episode at LTHT prior to their cancer diagnosis.

# **Results:**

| **Cancer Site** | **Cohort (n)** | **Diabetes Data Definition** | **HR (95% CI)** |
| --- | --- | --- | --- |
| All Cancers | LTHT Blood Catchment Area (29,098) | Clinical Coding | 1.18 (1.08-1.28) |
|  |  | Abnormal HbA1c | 0.76 (0.7-0.83) |
|  |  | Hybrid | 0.94 (0.87 - 1.02) |
|  | Patients with Prior Admissions (63,085) | Clinical Coding | 1.31 (1.26-1.36) |
|  |  | Abnormal HbA1c | 1.15 (1.10-1.19) |
|  |  | Hybrid | 1.19 (1.15-1.24) |
| Breast | LTHT Blood Catchment Area (4,670) | Clinical Coding | 1.36 (0.94) |
|  |  | Abnormal HbA1c | 0.66 (0.45-0.96) |
|  |  | Hybrid | 0.87 (0.63-1.21) |
|  | Patients with Prior Admissions (6,644) | Clinical Coding | 1.67 (1.4-1.99) |
|  |  | Abnormal HbA1c | 1.21 (1.03-1.41) |
|  |  | Hybrid | 1.28 (1.1-1.48) |
| Prostate | LTHT Blood Catchment Area (3,407) | Clinical Coding | 1.53 (1.08-2.18) |
|  |  | Abnormal HbA1c | 0.86 (0.6-1.21) |
|  |  | Hybrid | 1.06 (0.78-1.44) |
|  | Patients with Prior Admissions (5.604) | Clinical Coding | 1.57 (1.32-1.87) |
|  |  | Abnormal HbA1c | 1.38 (1.18-1.61) |
|  |  | Hybrid | 1.49 (1.28-1.71) |

Supplemental File 2 - Table 1: Cox derived hazard ratios for the relationship between diabetes and death for each diabetic data definition across the LTHT blood catchment area cohorts and patient population with prior admissions for all cancer patients, those with breast cancer and those with prostate cancer.

# **Discussion:**

The results of these further analyses add further ambiguity to the question of what the impact of diabetes mellitus is on the outcomes for patients with cancer.^3–6^ Each of the additional analysis cohorts is smaller than the main study cohorts which results in a corresponding decrease in the precision of the point estimates obtained.

Within the analyses undertaken focussed on the admitted cohort all results showed diabetes, irrespective of the cancer type or definition of diabetes, was associated with worse outcomes for patients with diabetes. The relative pattern of effect for each followed that found within the main analysis with the blood-based definition being the most optimistic, the coding being the most pessimistic and the hybrid definition being somewhere in the middle. The magnitude of effect also remained clinically meaningful and similar clinically to the results of the survival analysis undertaken in the larger population found within the main paper.

The results for the analysis within the LTHT blood catchment area population are however less consistent. Point estimates suggested that clinical coding identifies diabetic patients as having worse outcomes where the other definitions suggest a possible improvement in outcomes for patients with diabetes. Several of the analyses show that the effect is not unidirectional and therefore may represent no difference or results lacking sufficient precision for the scale of effect seen, if one were to exist.

It is important to note that all the survival analyses undertaken within this paper suffer with this issue of potential selection bias.^7^ By defining cohorts based on the presence or absence of certain features or characteristics those features are being conditioned on and may introduce a selection bias. This includes the presence or absence of an admission, geographical location, or the comorbidity of interest itself. Causal inference methods would suggest that this may result in misestimation of attributable risk such that results may be amplified, suppressed or even reversed. For example, patients with a prior admission may represent a sicker population than the background population but where the effect of this differs between patients with and without diabetes. In the case of the blood laboratory catchment area defined population, it is possible that difference between urban and rural populations may affect the results with worse outcomes having been shown previously in rural areas.^8–10^ This is similar to the issues seen in neonatal outcomes research which are well documented in the birth weight paradox.^11^ It is for this reason that the survival analysis within this publication is presented not as attempting to answer the question of the effect of diabetes on cancer outcomes but merely as a vehicle for demonstrating how poor or limited data definitions may significantly alter the estimates for subsequent analyses or prognostic tools which rely on them.

# **Conclusion:**

Across all survival analyses undertaken a consistent pattern was identified which was that the differing definitions of diabetes mellitus resulted in meaningful differences in outcome estimates. This adds further weight to the need for comprehensive data definitions and also the need for further causal inference methods-based analyses to attempt to address the underlying question of the impact of diabetes on cancer outcomes.

# **References:**

1. Cox DR. Regression Models and Life-Tables. *J R Stat Soc Ser B Methodol*. 1972;34(2):187-202. doi:10.1111/j.2517-6161.1972.tb00899.x

2. Deas I, Robson B, Wong C, Bradford M. Measuring Neighbourhood Deprivation: A Critique of the Index of Multiple Deprivation. *Environ Plan C Gov Policy*. 2003;21(6):883-903. doi:10.1068/c0240

3. Abdel-Rahman O. Impact of diabetes comorbidity on the efficacy and safety of FOLFOX first-line chemotherapy among patients with metastatic colorectal cancer: a pooled analysis of two phase-III studies. *Clin Transl Oncol Off Publ Fed Span Oncol Soc Natl Cancer Inst Mex*. 2019;21(4):512-518.

4. Akhavan S, Ghahghaei-Nezamabadi A, Modaresgilani M, et al. Impact of diabetes mellitus on epithelial ovarian cancer survival. *BMC Cancer*. 2018;18(1):1246.

5. Backemar L, Djarv T, Wikman A, et al. The role of diabetes and other co-morbidities on survival after esophageal cancer surgery in a population-based study. *Am J Surg*. 2013;206(4):539-543.

6. Amptoulach S, Gross G, Kalaitzakis E. Differential impact of obesity and diabetes mellitus on survival after liver resection for colorectal cancer metastases. *J Surg Res*. 2015;199(2):378-385.

7. Munafò MR, Tilling K, Taylor AE, Evans DM, Davey Smith G. Collider scope: when selection bias can substantially influence observed associations. *Int J Epidemiol*. 2018;47(1):226-235. doi:10.1093/ije/dyx206

8. Bhatia S, Landier W, Paskett ED, et al. Rural–Urban Disparities in Cancer Outcomes: Opportunities for Future Research. *JNCI J Natl Cancer Inst*. 2022;114(7):940-952. doi:10.1093/jnci/djac030

9. Clelland D, Hill C. Deprivation, policy and rurality: The limitations and applications of area-based deprivation indices in Scotland. *Local Econ*. 2019;34(1):33-50. doi:10.1177/0269094219827893

10. Murage P, Bachmann M, Jones A, Murchie P, Crawford M. Impact of travel time and rurality on presentation and outcomes of symptomatic colorectal cancer: A cross-sectional cohort study in primary care. *Br J Gen Pract*. 2017;67(660):e460-e466. doi:10.3399/bjgp17X691349

11. Hernandez-Diaz S, Schisterman EF, Hernan MA. The Birth Weight “Paradox” Uncovered? *Am J Epidemiol*. 2006;164(11):1115-1120. doi:10.1093/aje/kwj275
